# Interdisciplinary Therapy for patients with dementia

**DOI:** 10.1590/S1980-57642014DN83000013

**Published:** 2014

**Authors:** Maysa Luchesi Cera, Daniela Cristina Carvalho de Abreu, Rosângela de Abreu Venancio Tamanini, Amanda Carla Arnaut, Patrícia Pupin Mandrá, Carla da Silva Santana

**Affiliations:** 1 Speech Therapist at the CIRHER, Masters in Human Communication Disorders at the Federal University of São Paulo.; 2 Professor of Physiotherapy at the School of Medicine of Ribeirão Preto of the University of São Paulo (FMRP/USP).; 3 Occupational Therapist at the CIRHER. Masters of Science at the FMRP/USP.; 4 Physiotherapist at the CIRHER.; 5 Professor of Speech Therapy at the FMRP/USP.; 6 Professor of Occupational Therapy at the FMRP/USP.

**Keywords:** dementia, patient care team, interdisciplinary communication, therapy

## Abstract

Patients with dementia require rehabilitation involving several health professionals, where interdisciplinary care can further enhance the routine of patients and their families.

**Objective:**

To compare the functional performance of elderly with dementia before and after an interdisciplinary intervention program provided by a healthcare service of medium complexity.

**Methods:**

Three cases with clinically-confirmed dementia enrolled on an interdisciplinary rehabilitation program were reported. The following instruments were applied: Mini Mental-State Exam, Clinical Dementia Rating, Geriatric Depression Scale, Lawton & Brody Index, and the Functional Independence Measure for adults (FIM). The therapeutic strategies were individualized and designed based on patient performance on the FIM, according to the criteria of the Classification of Functionality, Disability and Health, implemented at the house of therapy of the Center for Integrated Rehabilitation together with provision of guidance.

**Results:**

A reduction in functional dependence was observed after intervention, evidenced by less supervision needed to carry out Activities of Daily Living.

**Conclusion:**

The three patients benefited from the interdisciplinary intervention.

## Abstract (pt)

Pacientes com demência necessitam de reabilitação com vários profissionais da saúde e a atenção interdisciplinar pode favorecer ainda mais a rotina do paciente e sua família.

**Objetivo:**

Comparar o desempenho funcional de idosos com demência antes e após um programa de estimulação interdisciplinar de um serviço de atenção à saúde de média complexidade.

**Métodos:**

Relato de três casos com diagnóstico médico de demência usuários de um programa de reabilitação interdisciplinar. Foram aplicados os instrumentos: Mini Exame do Estado Mental, *Clinical Dementia Rating, Geriatric Depression Scale*, Índice de Lawton & Brody e Medida de Independência Funcional para adultos (MIF). As estratégias terapêuticas foram individualizadas, delineadas a partir do desempenho na MIF, com base, nos domínios da Classificação Internacional de Funcionalidade, Incapacidade e Saúde, exercidas em uma casa terapêutica e associadas a orientações.

**Resultados:**

Após a intervenção, houve redução da dependência funcional, que evidencia diminuição da supervisão necessária para a realização das Atividades de Vida Diária.

**Conclusão:**

Os três pacientes se beneficiaram da intervenção interdisciplinar.

## INTRODUCTION

The growth in the elderly population has contributed to a greater prevalence of dementia. In 2005, an estimated 24 million people had dementia, a figure set to double every 20 years.^1^ Autopsies on dementia patients have revealed a 42% prevalence of Alzheimer's disease dementia (ADD), 23.5% vascular dementia, 21.5% mixed dementia (vascular and ADD) and 13% for other dementia types.^2^

Studies employing molecular biomarkers are exploring new diagnostic criteria and more advanced treatments,^3^ but current management of ADD is based on the use of anticholinesterases and memantine, according to systematic reviews.^4-7^ Utilization of interdisciplinary medical treatment has been described in recent years,^8-9^ as well as multidisciplinary non-medicamentous interventions, such as individualized longitudinal cognitive rehabilitation which augmented the effect of anticholinesterases in mild AD^10^ and programs promoting the practice of regular physical activity with a dual task allowing maintenance and improvement of cognitive and motor functions of elderly with ADD.^11,12^

In order to maintain and increase independence for performing Activities of Daily Living (ADLs), ADD patients require rehabilitation that involves several health professionals while interdisciplinary care can further enhance the routine of patients and their families. The effects of the disease go beyond cognitive impairments of the individual, impacting the family dynamic along with emotional and financial aspects of the family, making it difficult to provide the care needed to keep up ADLs, often leading to the caregivers discontinuing rehabilitation.

The Center for Integrated Rehabilitation of the State Hospital of Ribeirão Preto (CIRHER) handles cases of medium complexity from 26 municipal districts of the region and boasts gerontology that comprises physiotherapists, speech therapists, occupational therapists, a psychologist, a social worker, nursing assistant and an otolaryngologist (ENT specialist). Based on multidisciplinary rehabilitation of dementia patients, the CIRHER team has developed an individualized interdisciplinary therapy program drawing on the expertise of three areas of rehabilitation of this service (physiotherapy, speech therapy and occupational therapy) with the purpose of promoting the performance of ADLs. Therefore, the objective of this study was to compare the functional performance of elderly with dementia before and after an interdisciplinary intervention program provided by a secondary healthcare service.

## METHODS

This descriptive observational case report study was approved by the Research Ethics Committee of the School of Medicine of Ribeirão Preto of the University of São Paulo under protocol 1132/2008.

The three patients were referred to the CIRHER by physicians of a primary healthcare service, with diagnoses of probable ADD, according to the clinical criteria proposed by the Scientific Department of Cognitive Neurology and Aging of the Brazilian Academy of Neurology.^13^ The Clinical Dementia Rating (CDR) indicated different dementia stages among patients, including mild, moderate and severe dementia.

**Figure 1:**
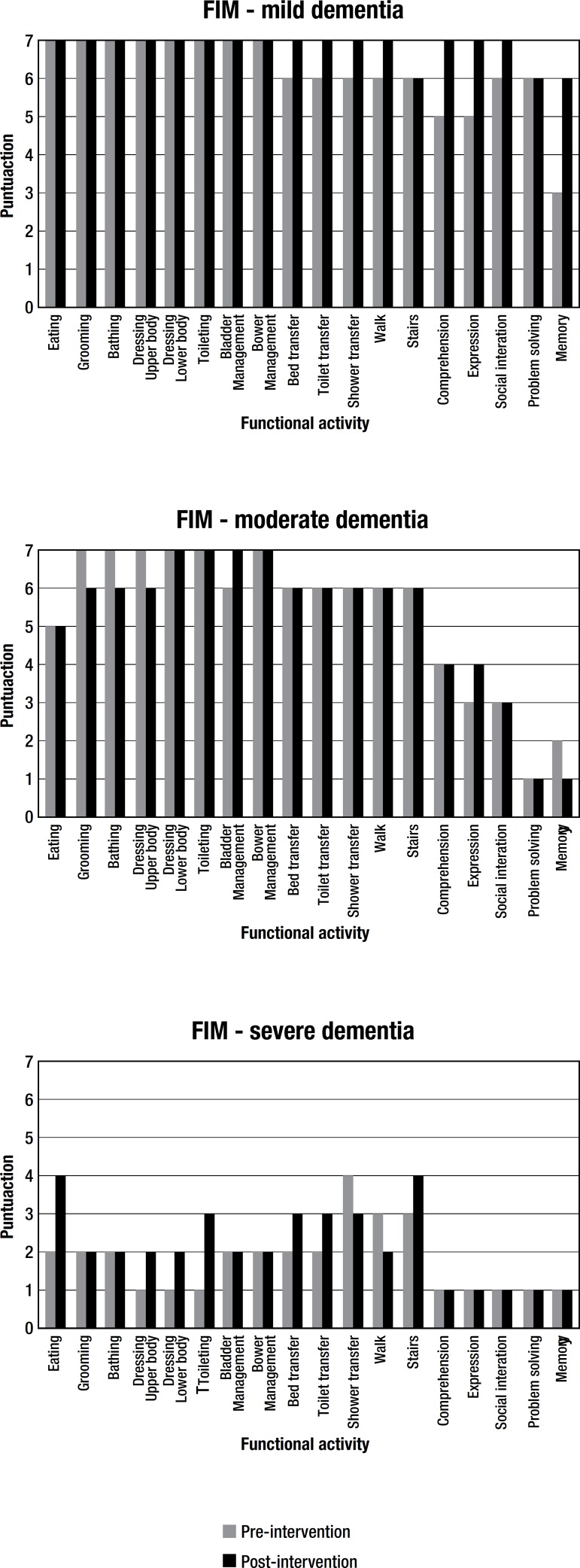
Results on Functional Independence Measure (FIM), pre and postintervention, for the three clinical cases assessed.

Prior to the intervention, all dementia patients underwent an initial 90-minute assessment performed during the first session by a member of the interdisciplinary team entailing the application of the following instruments: Mini-Mental State Examination - MMSE,^14,15^ for cognitive screening; CDR,^16,17^ for rating dementia severity; Geriatric Depression Scale (GDS),^18^ for detecting depressive symptoms; the Lawton & Brody Index (IADL),^19^ for assessing instrumental activities of daily living; and the Functional Independence Measure (FIM) for adults,^20^ to assess functional capacity, define the therapy program and follow-up patient evolution. The FIM provides a quantitative assessment of the burden of care required by an individual to perform a series of motor and cognitive activities of daily living.^20^ The FIM comprises 18 activities as depicted in Figure 1, each of which is scored from one to seven, where one point indicates full assistance and seven denotes full independence. The MMSE, CDR, GDS, IADL and FIM instruments were reapplied post intervention and before the last session of therapy.

The aim of the individualized interdisciplinary program was to improve functionality with the least supervision possible and greatest safety and was tailored according to each user's individual needs, based on their pre-intervention performance on the FIM. Therapeutic strategies were devised based on the criteria of the Classification of Functionality, Disability and Health. The length of the treatment varied according to the functional needs of each patient.

The 90-minutes sessions were held at the house of therapy of the CIRHE, a venue which replicates the domestic setting, and were given twice weekly to patients with mild or moderate dementia and once weekly to patients with severe dementia. A physiotherapist, speech therapist and an occupational therapist were present during all sessions but only one was the reference professional for the patient and responsible for giving commands and receiving instructions from the other therapists on the specifics of each area during activities.

Family were strategically involved in some sessions to ensure guidance was conveyed in a robust manner, with the exception of severe dementia patients, who were accompanied by caregivers at all times given their greater functional dependence. Based on the individualized stimulation, a Guidance Manual was produced and daily activities were suggested for family members and caregivers, who were concomitantly assisted by the service care (psychology and social service) team in a support group.

Table 1 shows examples of objectives, activities performed during therapy, and guidance given.

**Table 1:** Description of Intervention Objectives and Strategies according to the International Classification of Functionality, Disability and Health of the World Health Organization*.

ICF Constructs	Objectives	Strategies
Body functions and structures	• Compensate for impaired cognitive functions. Reduce behavioral alterations. • Reduce alterations in sensory (vestibular/balance) and neuromusculoskeletal functions. • Maintain safe ambulation as long as possible.	• Cognitive strategies: use of continued repetition of ADLs, emotional and logical context, spaced retrieval technique,22 training of problem solving, multimodal stimuli, guidance cards and signs, calendar, establishing routines; • Linguistic strategies: use of short utterances with simplified syntax, semantic categorization, tangible stimulus, allowing for adequate response delay.23 • Motor strategies: gait training (tandem, anterior, posterior and lateral); ascending and descending steps, circumventing obstacles, pivoting, transposition, sitting and standing, circuits, crouching down and elevating oneself to handle low or high objects, use of shin pads, dynadisc, stable and unstable surfaces and obstacles. For patients with severe dementia: only the most routine and highly contextualized activities were applied (anterior gait, sitting and standing, ascending and descending steps and circumventing obstacles).
Activities and participation	Reduce dependence in ADLs. • Decrease the restriction in functional and social participation.	• Promoting performance of self-care tasks (washing, personal hygiene, eating, dressing and looking after one’s health) through training and least supervision possible. • Organizing the domestic environment for safety (preparation of meals and other domestic tasks). • Semantically categorizing supermarket purchases or items of clothing. • Example items from the guidance manual: • In the morning, after breakfast, restore time bearings by referencing the calendar and, for patients with severe dementia, by checking daylight. • Involving individual in domestic activity by asking them to fetch a pack of rice from the pantry or a given garment from the clothes line and fold it. • Providing verbal command for any instruction: address them by name and use the core phrases ("Name, bend your legs and pick up the bowl"). Repeat the phrase or key word when necessary ("Bend your legs" or "Legs"). Use of visual cues only when necessary (point or provide example). • Establishing routine (schedule was devised with caregivers and placed in each patient’s Manual).
Environmental Factors	• Focus on physical environment. • Focus on the attitudinal environment in relation to patient performance. • Increase communication aids. • Facilitate comprehension of verbal stimuli. • Encourage turn-taking and spoken discourse with interlocutor.	• Organizing the environment to provide supervision (cues or materials, such as medication organizers); • Based on guidance instructions, adapt the structure of the home environment (lighting, placement of furniture, support rails). • Apply the cognitive, communication and motor strategies outlined above.

* International Classification of Functioning, Disability and Health of the World Health Organization (2003).^21^

## RESULTS

Patients were female, right-handed, widows with preserved gait, living with a son or daughter, and in regular use of anticholinesterases. The one patient with severe dementia had two caregivers and was also in use of Memantine. Table 2 shows the sociodemographics and clinical data of the patients. The number of therapy sessions shown in Table 2 excludes the evaluation sessions. The patient with mild dementia missed some therapy sessions due to a medical examination. The other patients had absences because the caregiver had problems convincing them to leave home or because the caregiver was unable to accompany them.

**Table 2:** Sociodemographics and clinical characteristics of patients before and after intervention.

Variables	Patient 1	Patient 2	Patient 3
Age (years)	73	82	81
Schooling (years)	5	8	12
Onset of symptoms	2008	2007	2008
Diagnosis	2009	2012	2011
Start of therapy	June 2013	October 2012	March 2013
CDR* before	1	2	3
CDR* after	1	2	3
MMSE+ before	23	15	NA**
MMSE+ after	26	11	NA**
GDS§ before	4	2	NA**
GDS§ after	4	3	NA**
IADL** before	13	17	27
IADL** after	13	18	27
FIM^¶^ before	111	89	34
FIM^¶^ after	123	97	39
Duration of therapy (weeks)	8	19	13
Therapy sessions received	15 of 18	19 of 26	9 of 13
Conduct	Discharge	Discharge	Discharge

* CDR: Clinical Dementia Rating, 1 indicates mild , 2 moderate and 3 severe dementia. +MMSE: Mini-Mental State Examination, possible range: 0-30, higher scores indicating better performance; in individuals with 5 years or more of schooling, scores under 26 indicate impairment, according to Brucki et al. (2003).15 §GDS: Geriatric Depression Scale, possible range: 0-15, higher scores indicating worse performance, five points suggest alteration;

** IADL: Lawton & Brody Index, possible range: 0-27, higher scores indicating worse performance and two points suggests alteration.

¶ FIM: Functional Independence Measure, possible range: 18-126, higher scores indicating better performance; score of 18-36 indicates high level of dependence, 37-90 moderate dependence and 91-126 independence.

** NA: not applicable.

Figure 1 depicts the FIM results at pre and post-intervention for the three clinical cases treated.

Upon conclusion of the proposed intervention, all of the patients were discharged from the medium complexity healthcare service.

## DISCUSSION

The results of the FIM (Table 2 and Figure 1) reveal the benefits of the interdisciplinary intervention for the three patients followed, evidenced by the improvement on 17 items and maintenance on 31 items, out of the 54 functional abilities assessed by the instrument. The reduction in functional dependence demonstrated that the interdisciplinary therapy reduced the burden of supervision needed to carry out ADLs. Akin to a previous multiprofessional cognitive and functional rehabilitation program involving 24 sessions given over six months,^24^ the results of the present study showed stabilization and slight improvements in cognitive performance and ADLs. In a previous study, after 12 months of psychosocial intervention, three patients diagnosed with ADD demonstrated cognitive enhancement, functional stabilization and improvement of behavioral problems, but the benefits were not sustained throughout the second year of intervention.^25^ In our study, the mild dementia patient showed functional improvement within eight weeks and also a better score on cognitive screening, results indicating that the goals in this program can be attained within a relatively short intervention timeframe.

The patient with moderate dementia followed for the longest period had four of the six functional declines observed among the 54 functional abilities studied, i.e. the benefits from applying adequate supervision in patient performance appear in the first few months of the intervention yet functional declines emerge in less than six months. As illustrated in Figure 1, functional decline of the patient occurred for functions associated with a high cognitive demand and was consistent with the decline observed on cognitive screening (Table 2). Robust changes in cognition are unlikely to occur as a consequence of cognitive training in patients with ADD,^26^ and the main goal of rehabilitation is therefore to define the most effective supervision strategies to enable individuals to maximize the use of their functionality potential. In the absence of rehabilitation, the patient may have exhibited even worse decline. Given that the objectives set were achieved in eight weeks, it follows that functional reassessment should be carried out between the 8^th^ and 12^th^ weeks to define future conduct: continuation of the therapy or discharge with follow-up in primary healthcare.

For the severe dementia case, the supervision strategies were devised to offer greater physical safety and reduce fall risk. Results showed worsening on only two items (Figure 1). Although studies report benefits of rehabilitation in mild and moderate dementia,^10,25,26^ our results showed benefits of the therapy even in severe ADD, promoting maintenance and improvement of functionality.

Intervention results observed in FIM scores were not evident on the IADL. FIM was more sensitive for detecting therapeutic responses probably because it specifies the basic activities of daily life. By contrast, the IADL specifies dependence on an instrumental activity that involves complex tasks. The IADL scores indicate the benefit of intervention in maintaining the skills of the subjects in performing complex tasks.

From the language strategies applied, it was evident that the communication approach adopted with patients was essential in allowing the most effective supervision to be applied by both professionals and caregivers. According to FIM scores, patients' communication performance was maintained and enhanced.

Mirroring the observations by Lang et al.,^8^ we found that interdisciplinary treatment promoted adequate and effective communication among team members and consequent selection of the optimal treatment strategy. Sharing the load with other disciplines allows each to bring its background, experience, and expertise to bear on the needs of the patient and to facilitate optimal long-term outcomes.^27^ In addition, interdisciplinary rehabilitation requires lower investment in terms of time spent per week when compared to multidisciplinary approaches and consequently reduces overload on the family routine and aids in maintaining the patient's wake state. Moreover, the functional interdisciplinary activities were conducive for the participation of caregivers who received more robust guidance for the specific ADLs and not according to the therapeutic specialty (physiotherapy, occupational therapy or speech therapy). Thus, the therapy applied during the sessions was more easily integrated into the patient's routine, thereby improving the functionality of the intervention despite its inherently shorter duration and length of patient stay in the service.

Interdisciplinary team members have areas of practice that overlap so team leadership is enhanced when the members understand each other's contributions and expertise.^27^ Our program was found to facilitate the performance of activities by the patients while also promoting professional development among the therapists involved, in as far as the strategies adopted by one specialist were subsequently taken up by the others, including application to other patients with similar needs. It is important to emphasize that further studies involving a larger number of patients are needed to measure the effect of treatment and also to allow comparison between multidisciplinary and interdisciplinary programs.

Changes in the functionality of patients with dementia could be evaluated qualitatively and quantitatively through the instruments selected. However, changes in functionality status of the patient also have an impact on work and perception by the caregiver. It is thus suggested that instruments for assessing burden can also be included with those used in this study.

Therefore, the interdisciplinary intervention yielded functional benefits for patients with ADD and promoted professional growth among the rehabilitation team involved.
